# IKKi Deficiency Promotes Pressure Overload-Induced Cardiac Hypertrophy and Fibrosis

**DOI:** 10.1371/journal.pone.0053412

**Published:** 2013-01-22

**Authors:** Jia Dai, Di-Fei Shen, Zhou-Yan Bian, Heng Zhou, Hua-Wen Gan, Jing Zong, Wei Deng, Yuan Yuan, FangFang Li, Qing-Qing Wu, Lu Gao, Rui Zhang, Zhen-Guo Ma, Hong-Liang Li, Qi-Zhu Tang

**Affiliations:** 1 Department of Cardiology, Renmin Hospital of Wuhan University, Wuhan, China; 2 Cardiovascular Research Institute of Wuhan University, Wuhan, China; 3 Department of Cardiology, Institute of Cardiovascular Disease, Union Hospital, Tongji Medical College, Huazhong University of Science and Technology, Wuhan, People's Republic of China; Ohio State University Medical Center, United States of America

## Abstract

The inducible IκB kinase (IKKi/IKKε) is a recently described serine-threonine IKK-related kinase. Previous studies have reported the role of IKKi in infectious diseases and cancer. However, its role in the cardiac response to pressure overload remains elusive. In this study, we investigated the effects of IKKi deficiency on the development of pathological cardiac hypertrophy using in vitro and in vivo models. First, we developed mouse models of pressure overload cardiac hypertrophy induced by pressure overload using aortic banding (AB). Four weeks after AB, cardiac function was then assessed through echocardiographic and hemodynamic measurements. Western blotting, real-time PCR and histological analyses were used to assess the pathological and molecular mechanisms. We observed that IKKi-deficient mice showed significantly enhanced cardiac hypertrophy, cardiac dysfunction, apoptosis and fibrosis compared with WT mice. Furthermore, we recently revealed that the IKKi-deficient mice spontaneously develop cardiac hypertrophy. Moreover, in vivo experiments showed that IKKi deficiency-induced cardiac hypertrophy was associated with the activation of the AKT and NF-κB signaling pathway in response to AB. In cultured cells, IKKi overexpression suppressed the activation of this pathway. In conclusion, we demonstrate that IKKi deficiency exacerbates cardiac hypertrophy by regulating the AKT and NF-κB signaling pathway.

## Introduction

Heart failure (HF) is a debilitating disease with a high prevalence, morbidity and mortality [1], [2], [3], [4], [5]. Pathological cardiac hypertrophy is an important predecessor of heart failure that is characterized by cardiac dysfunction, cell enlargement, reactivation of foetal gene expression, impaired myocardial vascularization, phenotypic changes in the extracellular matrix and hyperplasia of fibrosis [6], [7], [8], [9]. Recent studies have shown that signaling pathways and their associated molecules play complex and pivotal roles in the development of cardiac hypertrophy,including mitogen activated protein kinases (MAPKs), phosphatidylinositol 3-kinase(PI3K)/AKT and calcineurin/nuclear factor of activated T cells (NFAT) [10]. However, effective blockade of the hypertrophy and prevention of transition to congestive heart failure remain a challenge. Thus, the identification of signals and pathways involved in pathological hypertrophy would open the door for the development of future therapeutic interventions for heart failure.

Nuclear factor-κB (NF-κB) plays a critical role in the immune response and influences gene expression events that affect cell survival, apoptosis, differentiation, proliferation, cancer progression and development [11], [12]. The NF-κB family of transcription factors includes five members, p50, p52, p65 (RelA), c-Rel, and RelB. These members share an N-terminal Rel homology domain (RHD),which is responsible for DNA binding and homo- and heterodimerization [11], [12]. In the absence of a stimulus, NF-κB dimers normally combine with one of three typical IκB proteins, IκBα, IκBβ or IκBε, or the precursor protein p100. Stimulation with cytokines or other agonists results in the phosphorylation of IκB by the inhibitory-κB kinase (IKK) complex, which includes IKKα, IKKβ and IKKγ, triggering the degradation of IκB. Then, the freed NF-κB translocates to the nucleus, where it binds to and activates the promoters of the NFκB responsive genes [11], [12].

Recent studies have shown that NF-κB directly exerts its role or alternatively involved in G protein-coupled receptor agonist- or tumour necrosis factor α(TNFα)-induced cardiac hypertrophy and pathological remodeling and fibrosis and NF-κB inhibition attenuates cardiac hypertrophy [13], [14], [15], [16], [17]. Furthermore, IKKβ-deficient mice exhibit cardiac dilation and dysfunction and lung congestion [18]. The inducible IκB kinase (IKKi/IKKε) a constitutively active serine-threonine IKK-related kinase shares 31% amino acid identity with IKKβ in the highly conserved N-terminal kinase domain but differs from IKKβ in several important aspects [19]. For example, IKKi is expressed in the cells and tissues of the immune system [19]. Recent studies have shown that human IKKi has two novel splice variants, IKKε-sv1 and IKKε-sv2, which have cell type- and stimulus-specific protein expression [20]. Some groups have described a role for IKKi in infectious diseases and cancer [21], [22], [23], [24], [25], [26]. However, it has not been shown to be involved in cardiovascular disease. In this study, for the first time, we used IKKi-knockout (KO) mice to investigate the role of IKKi in cardiac hypertrophy induced by pressure overload. We demonstrate that IKKi deficiency in mice leads to cardiac hypertrophy, fibrosis, and cardiac dysfunction, indicating a crucial role for IKKi in regulating cardiac hypertrophy.

## Materials and Methods

### Animals and animal models

All animal procedures were performed in accordance with the *Guide for the Care and Use of Laboratory Animals*, which was published by the U.S. National Institutes of Health (NIH Publication No. 85-23, revised 1996) and approved by the Animal Care and Use Committee of the Renmin Hospital of Wuhan University (protocol number: 00020390).Male KO mice(C57BL/6 background; knockout mice were purchased from Jackson Laboratory, No. 006908) and their wild-type (WT) littermates, which ranged in age from 8 to 10 weeks, were subjected to aortic banding (AB) as described previously [27]. Each mouse was were anaesthetized with sodium pentobarbital (Sigma, 80 mg/kg, ip), and a horizontal skin incision was made at the level of 2–3 intercostal space. The descending aorta was isolated, and a 7.0 silk suture was wrapped around the aorta.A bent 26-gauge needle (for 25.5–27.5 g) or 27-gauge needle (for 23.5–25.5 g) was then placed next to the aorta, and the suture was tied snugly around the needle and the aorta. After ligation, the needle was quickly removed, the chest and skin were closed, and the mice were allowed to recover. Sham-operated mice underwent the same procedure without constriction. The adequacy of anesthesia was monitored during the surgical procedures by assessing the lack of the pedal withdrawal reflex, slow constant breathing, and no response to surgical manipulation. Buprenorphine (0.1 mg/kg, sc) was administered for post-operative analgesia. Four weeks after the operation, the hearts, lungs, and tibiae of the mice were dissected and weighed or measured to compare the heart weight (HW)/body weight (BW) (mg/g), HW/tibial length (TL) (mg/mm), and lung weight (LW)/BW (mg/g) ratios of the different groups.

### Echocardiography and hemodynamic analyses

Echocardiography was performed on anesthetized (1.5% isoflurane) mice using a MyLab 30CV (ESAOTE S. P. A) with a 15 MHz linear array ultrasound transducer. The left ventricle (LV) dimensions were assessed in the parasternal short-axis view. End-systole and end-diastole were defined as the phases that were associated with the smallest and largest areas of the LV, respectively. The end-systolic (LVESD) and end-diastolic (LVEDD) LV internal diameters and posterior wall end-diastolic thickness (PWT) were measured from the LV M-mode tracing with a sweep speed of 50 mm/s at the mid-papillary muscle level. The percentage of fractional shortening (FS) was calculated as (LVEDD-LVESD)/LVEDD×100.

To perform the invasive hemodynamic measurements, we anesthetized the mice with 1.5% isoflurane, and a microtip transducer catheter (SPR-839, Millar Instruments, Houston, TX, USA) was inserted into the right carotid artery and advanced into the left ventricle. The pressure signals and heart rates were recorded continuously using a Millar Pressure-Volume System (MPVS-400, Millar Instruments, Houston, TX, USA), and the recordings were further analyzed using PVAN data analysis software.

### Histological analysis and apoptotic cell assay

The hearts were excised, washed with PBS, arrested in diastole with 10% potassium chloride solution, weighed, placed in 10% formalin, and embedded in paraffin. They were then cut transversely and close to the apex to visualize the left and right ventricles. Several sections of each heart (4–5 µm thick) were prepared, stained with hematoxylin and eosin (H&E) for histopathology or picrosirius red (PSR) for collagen deposition by standard procedures and then visualized by light microscopy. For the myocyte cross-sectional area, sections were stained for membranes with FITC-conjugated WGA (Invitrogen) and for nuclei with DAPI. Single myocytes were measured using a quantitative digital image analysis system (Image Pro-Plus, version 6.0). The outlines of 100 myocytes were traced for each group. Cell death by apoptosis was evaluated using a TUNEL assay, which was performed on sections with In Situ Apoptosis Detection Kit (Roche, 11684817910) according to the manufacturer's recommendations.

### Quantitative real-time RT-PCR

Real-time PCR was performed to detect the mRNA expression levels of hypertrophic and fibrotic markers. Total RNA was extracted from frozen pulverized mouse cardiac tissue using TRIzol (Invitrogen, 15596-026). The yields and purities were spectrophotometrically estimated using the A260/A280 and A230/260 ratios, as measured with a SmartSpec Plus Spectrophotometer (Bio-Rad). The RNA (2 µg of each sample) was reverse-transcribed into cDNA using oligo(DT) primers and the Transcriptor First Strand cDNA Synthesis Kit (Roche, 04896866001). The PCR amplifications were quantified using the LightCycler 480 SYBR Green 1 Master Mix (Roche,04707516001). The results were normalized against glyceraldehyde-3-phosphate dehydrogenase (GAPDH) gene expression.

### Protein extraction and western blotting analyses

The left ventricles were harvested for western blotting analyses. They were first lysed in RIPA lysis buffer, and the protein concentrations were measured using the BCA Protein Assay Kit (Thermo, 23227) and an ELISA Reader(Synergy HT, Bio-Tek). The cell lysates (50 µg) were loaded into each lane and subjected to SDS-PAGE, and the proteins were then transferred onto Immobilon-FL transfer membranes (Millipore, IPFL00010). The membranes were incubated overnight at 4°C with primary antibodies against one of the following proteins: p-MEK1/2 (Cat#, 9154), T-MEK1/2 (Cat#,9122), p-ERK1/2 (Cat#,4370), T-ERK1/2 (Cat#,4695), p-P38 (Cat#,4511), T-P38 (Cat#,9212), p-JNK(Cat#,4668),T-JNK (Cat#,9258), p-PI3K(Cat#, 4228), T-PI3K (Cat#,4257), p-AKT (Cat#,4060), T-AKT (Cat#,4691), P-GSK3β (Cat#,9322), T-GSK3β (Cat#,9315), p-mTOR (Cat#,2971), T-mTOR (Cat#,2983), P-FOXO3A (Cat#,9465), T-FOXO3A (Cat#,2497), P-FOXO1 (Cat#,9461), T-FOXO1 (Cat#,2880), p-NFκB (Cat#,3033), T-NFκB (Cat#,4764), Bax (Cat#,2772), Bcl2(Cat#, 2870),Cleaved Caspase3(Cat#,9661),GAPDH(Cat#,2118),and IKKi(Cat#,3416). All antibodies were purchased from Cell Signaling Technology.The membranes were then incubated with goat anti-rabbit IgG (LI-COR, 926-32211) for one hour IgG. The blots were scanned using a two-color infrared imaging system (Odyssey, LI-COR). Specific protein expression levels were normalized to the GAPDH protein for the total cell lysates and cytosolic proteins.

### H9c2 cell culture and surface area

Cultures of H9c2 rat cardiomyocyte cells (ATCC, Rockville, MD, USA) were prepared as described previously [28]. H9c2 cells were seeded at a density of 1×10^6^cells/well onto 6-well culture plates in Dulbecco's modified Eagle's mediu-m (DMEM)/F12 1∶1 medium mixed at a ratio of 1∶1 (v/v) (Gibco,C11995) with 10% fetal bovine serum (FBS;Gibco,1133067), glutamine (2 mmol/L), penicillin (100 IU/ml), and streptomycin (100 mg/ml). After 48 hours, the culture medium was replaced with F10 medium containing 0.1% FBS,and the cells were infected with different adenoviruses followed by angiotensin II (Ang II; 1 µM) treatment.For the cell infections,cardiac myocytes were cultured in 6-well plates at a density of 1×10^6^ cells/well and then exposed to 2×10^8^ pfu of each virus in 1 ml of serum-free medium for 24 hours.The cells were then washed and incubated in serum-containing medium for 24 hours.The virus Ad-IKKi was used to overexpress IKKi, and the control virus AdGFP was used as a control.

To identify the cardiomyocytes and assess cardiomyocyte hypertrophy, we characterized the cells by analyzing their cardiac α-actinin expression using immunofluorescence.The cells were washed with PBS, fixed with RCL2 (ALPHELYS, RCL2-CS24L), permeabilized in 0.1%Triton X-100 in PBS, and stained with anti-α-actinin (Millipore, 05-384) at a dilution of 1∶100 in 1% goat serum. The secondary antibody was Alexa Fluor® 488 goat anti-mouse IgG (Invitrogen, A11004). The myocytes on coverslips were mounted onto glass slides with SlowFade Gold antifade reagent with DAPI (Invitrogen, S36939).

### Statistical analysis

Data are expressed as the means ± SEM. Differences among the groups were determined by a two-way ANOVA followed by a post hoc Tukey's test. Comparisons between two groups were performed using an unpaired Student's t-test. A p-value of <0.05 was considered to be statistically significant.

## Results

### IKKi expression is induced in hypertrophic hearts following AB

**Figure 1.IKKi:**
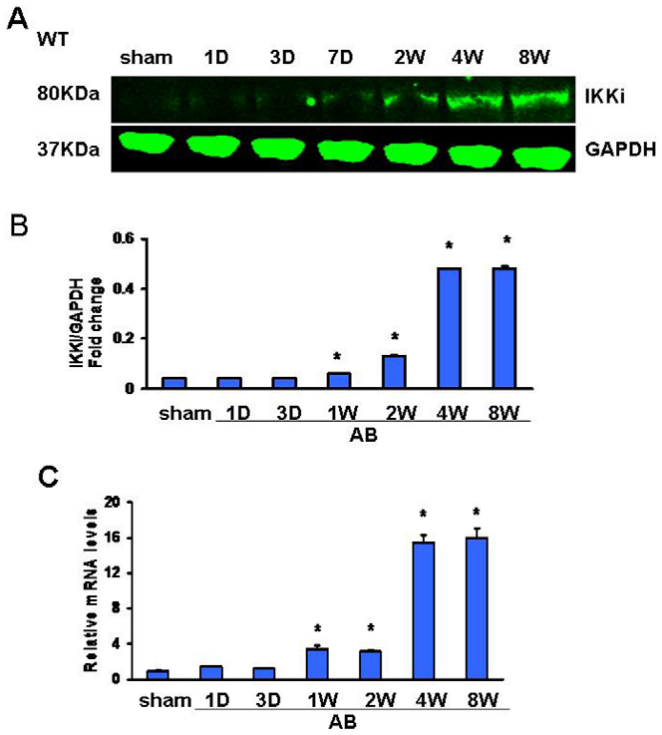
IKKi expression in the hypertrophic heart. A,Western blot analysis of the cardiac IKKi protein in WT mice after aortic banding at the time points indicated (n = 6). B, RT-PCR analysis of cardiac IKKi mRNA levels in WT mice after aortic banding at the time points indicated (n = 6). *P<0.05 vs. sham.

To investigate the potential role of IKKi in cardiac hypertrophy, we used the well-established cardiac hypertrophy model induced by AB. We found that IKKi protein and mRNA levels were slightly increased at 1 week but significantly up-regulated at 4 and 8 weeks after AB (Figure 1). These findings demonstrate that IKKi expression compensatorily increases during the development of cardiac hypertrophy.

### IKKi deficiency induces severe and spontaneous hypertrophy

To evaluate alterations in cardiac structures and functions in IKKi-deficient mice, we harvested the hearts of 30-week-old mice and performed echocardiograms. It was documented that the HW, HW/BW, HW/TL, LVEDD, and LVESD were significantly increased and the FS was decreased compared with the WT mice (Table 1), which agrees with our hypothesis that the loss of IKKi leads to spontaneous cardiac hypertrophy and dysfunction.

**Table 1:** Anatomic and echocardiographic analysis of 24- to 30 -week-old IKKi KO mice and WT mice.

Parameter	WT(n = 6)	IKKi KO(n = 6)
BW (g)	30.39±0.66	31.08±0.73
HW/BW(mg/g)	4.05±0.09	5.43±0.22*
LW/BW(mg/g)	4.67±0.15	4.44±0.16
HW/TL(mg/cm)	6.54±0.24	9.10±0.32*
HR (beats/min)	501±12	520±23
LVEDD(mm)	3.53±0.05	4.25±0.11*
LVESD(mm)	2.08±0.06	2.78±0.12*
LVPWD (mm)	0.72±0.01	0.73±0.02*
FS (%)	41.22±1.28	34±1.18*

BW,body weight;HW/BW,heart weight/body weight;LW/BW,lung weight/body weight; HW/TL,heart weight/tibial length; HR,heart rate; LVEDD,left ventricular end-diastolic dimension; LVESD,left ventricular end-systolic diameter; LVPWD,left ventricular posterior wall dimension; IVSD, Interventricular septal thickness at end-diastole; FS,fractional shortening.

* *P<0.05* vs WT/KO.

### IKKi deficiency enhances cardiac hypertrophic and dysfunctional responses to pressure overload

**Figure 2:**
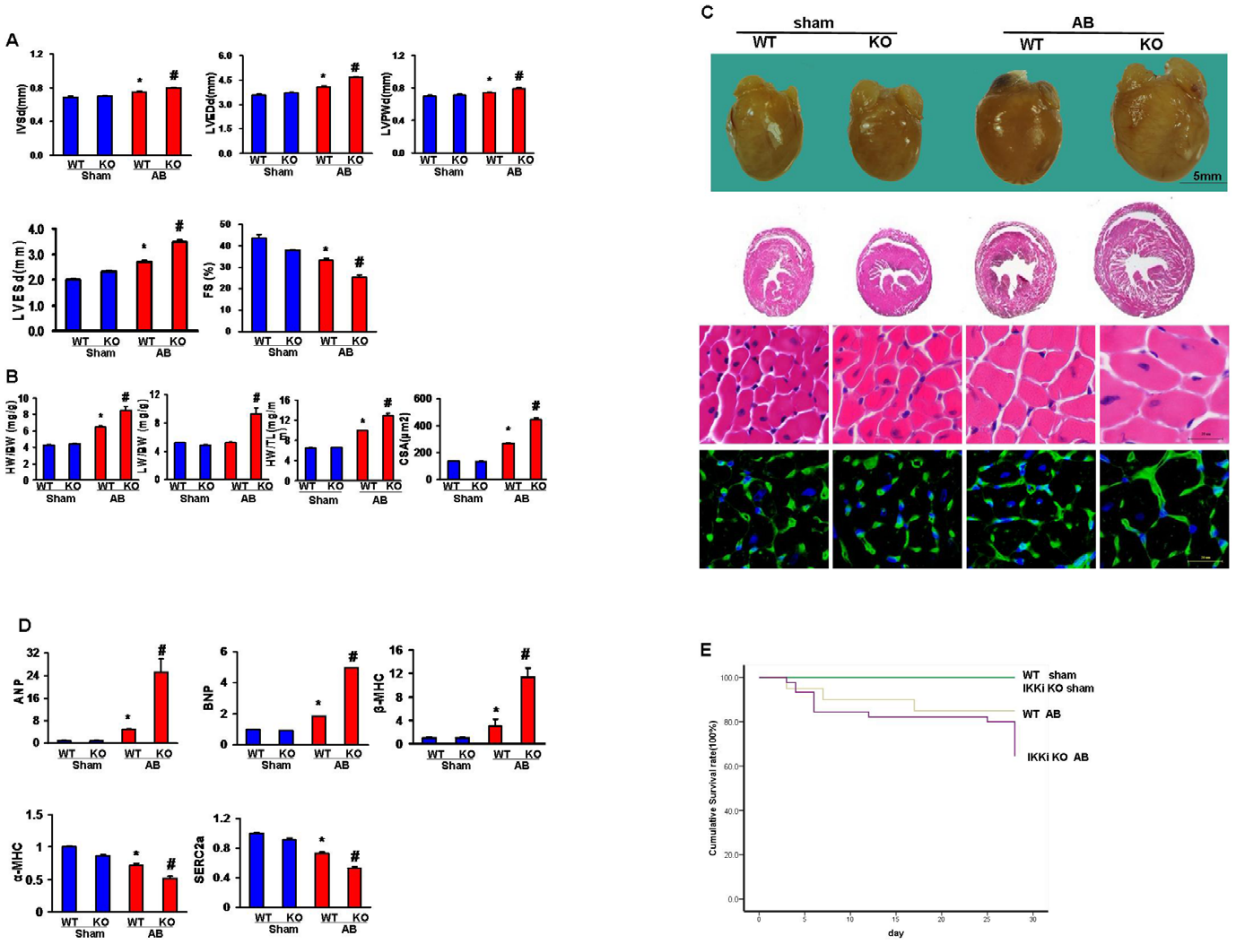
Effects of IKKi on cardiac hypertrophy. A, Echocardiographic results for the 4 groups of mice at 4 weeks following AB or sham surgery (n = 6). B, Statistical results of the HW/BW, LW/BW, and HW/TL ratios and myocyte cross-sectional areas of the indicated groups. C, Gross hearts, HE staining and WGA-FITC staining of the sham and AB mice at 4 weeks post-surgery. D, Expression levels of the transcripts of ANP, BNP, β-MHC, α-MHC and SERCA2α after AB were determined by RT-PCR analysis (n = 6). E. Kaplan-Meier curve depicting the survival of WT-sham, KO-sham, WT-AB, and KO-AB mice *P<0.05 vs. WT/sham. # *P*<0.05 vs. WT/AB following AB.

To clarify the direct relationship between IKKi deficiency-mediated changes and cardiac hypertrophy, IKKi-KO mice and their WT littermates were subjected to cardiac pressure overload by AB or a sham surgery. The cumulative survival rate at 4 weeks after AB was strikingly decreased by IKKi deficiency (Figure 2E). Echocardiographic analyses were also utilized to evaluate cardiac structures and functions, including the chamber diameter, wall thicknesses and function of the left ventricle. The KO and WT mice that underwent sham surgery did not differ echocardiographically. However, the echocardiographic measurements of LVEDD, LVESD, interventricular septal thickness at end-diastole (IVSD), left ventricular posterior wall thickness at end-diastole (LVPWD), and fractional shortening (FS) indicated deteriorated cardiac hypertrophy and dysfunction in the KO mice compared with the WT mice (Figure 2A). The LV hemodynamic parameters of the anesthetized mice that were obtained during the acquisition of the pressure-volume (PV) loop further confirmed this significantly deteriorated hemodynamic dysfunction (volume and systolic and diastolic function) of the LV in the IKKi-KO mice as shown in Table 2. Under basal conditions, pressure-overloaded KO mice showed significantly increased ratios of HW/BW, HW/TL and LW/BW and cardiomyocyte cross-sectional area (CSA) compared with the WT mice. No significant differences were observed between the sham-operated groups (Figure 2B). The gross hearts and H&E and WGA staining results were also consistent with the role of IKKi as an inhibitor of cardiac hypertrophy (Figure 2C). Furthermore, real-time PCR was performed to analyze the mRNA expression of hypertrophic markers (Figure 2D). As expected, we found significantly higher expression levels of the cardiac fetal genes atrial natriuretic peptide (ANP), B-type natriuretic peptide (BNP), and β-myosin heavy chain (β-MHC) in the KO mice in response to AB. However, the mRNA expression levels of α-myosin heavy chain (α-MHC) and sarcoendoplasmic reticulum Ca^2+^-ATPase (SERCA2α) were reduced. These results indicate that IKKi deficiency is responsible for the progressive hypertrophic effects and the impairment of cardiac function that is induced by pressure overload.

**Table 2:** Anatomic and hemodynamic parameters in IKKi KO and WT mice at 4 weeks after surgery.

Parameter	Sham		AB	
	WT(n = 6)	IKKi KO(n = 6)	WT(n = 6)	IKKi KO(n = 6)
BW (g)	27.50±0.52	27.19±0.32	27.89±0.34	28.02±0.40
HW/BW(mg/g)	4.29±0.07	4.43±0.04	6.49±0.08*	8.53±0.42* #
LW/BW(mg/g)	5.20±0.05	4.90±0.10	5.19±0.13	9.33±0.76* #
HW/TL(mg/cm)	6.40±0.13	6.51±0.08	9.96±0.08*	12.83±0.48* #
HR (beats/min)	483±9	462±10	477±8	462±18
ESP (mmHg)	105.74±1.58	117.15±4.04	150.85±2.16*	149.25±2.30*
EDP (mmHg)	9.84±0.19	10.24±1.28	17.75±2.14*	24.31±1.84* #
ESV (ml)	10.09±0.44	10.51±1.46	23.58±1.93*	37.23±3.46* #
EDV (ml)	26.82±0.78	24.85±1.83	34.44±1.32*	48.37±3.29* #
dP/dt max (mmHg/s)	10585.47±540.98	10663.83±781.97	8171.17±326.82*	6694.33±306.38* #
dP/dt min (mmHg/s)	−9177.34±269.63	−8967.33±357.21	−7658.17±346.37*	−6578.17±416.63* #
EF(%)	65.60±1.35	64.02±2.66	39.18±2.40*	25.83±3.11* #

BW,body weight;HW/BW,heart weight/body weight;LW/BW,lung weight/body weight; HW/TL,heart weight/tibial length; HR,heart rate; ESP, end-systolic pressure; EDP, end-diastolic pressure; ESV, endsystolic volume; EDV, end-diastolic volume; EF, ejection fraction; dP/dtmax, maximal rate of pressure development; dP/dtmin, maximal rate of pressure decay.

* *P<0.05* vs WT/sham;

# *P<0.05* vs WT/AB after AB.

### Forced IKKi expression attenuates the hypertrophic growth of myocytes in vitro

**Figure 3:**
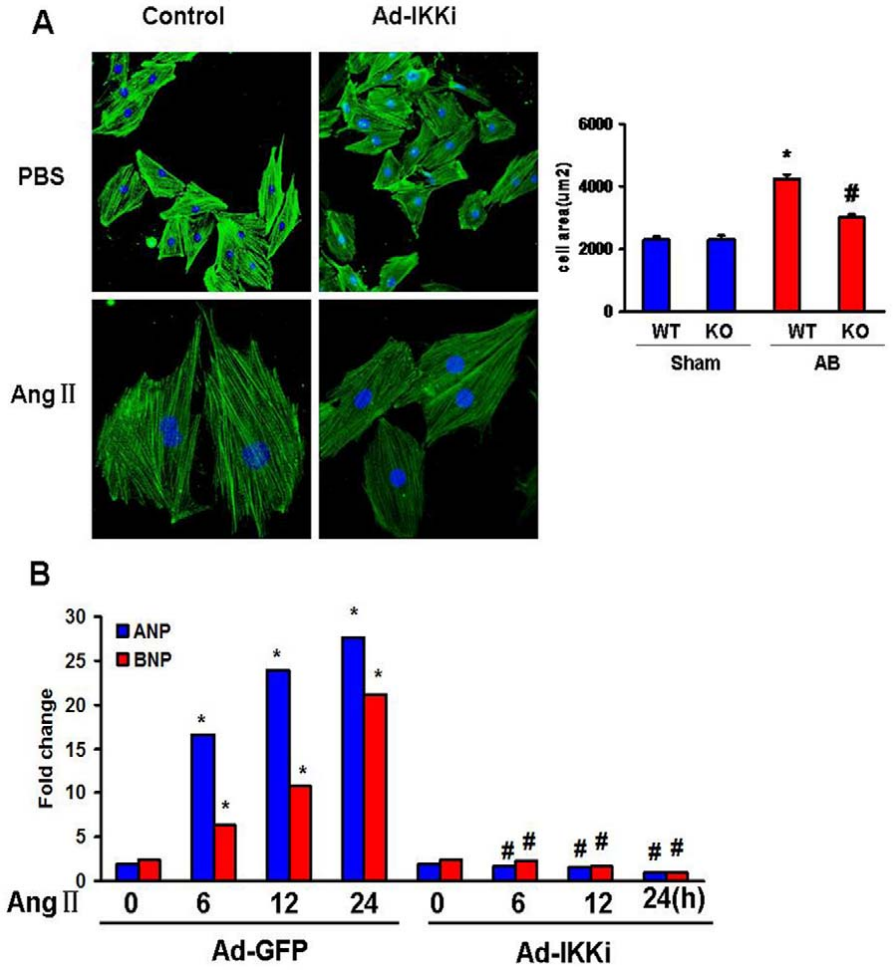
IKKi overexpression attenuates myocyte hypertrophy in vitro. A, The inhibitory effect of IKKi overexpression on the enlargement of myocyte induced by Ang II (1 µM for 48 h). B, RT-PCR analysis of the mRNA levels of ANP and BNP induced by Ang II at the time points indicated. * *P*<0.05 vs WT at the 0 time point. # *P*<0.05 vs WT at the same time point.

To specifically examine the role of IKKi in cardiomyocytes, we performed gain- of-function studies using cultured H9c2 rat cardiomyocytes. The H9c2 cells were serum-starved for 24 h in 0.5% FBS after infection with Ad-IKKi and then treated with 1 µM Ang II for the indicated times. Ad-IKKi infection led to a substantial increase in the level of IKKi protein in H9c2 rat cardiomyocytes. Further studies showed that the IKKi overexpression induced by Ad-IKKi infection attenuated Ang II-mediated cardiomyocyte hypertrophy, as measured by the cell surface area (Figure 3A). Moreover, RT-PCR showed that IKKi overexpression markedly decreased the mRNA levels of ANP and BNP induced by Ang II (Figure 3B). These in vitro data suggest the inhibitory effect of IKKi on cardiomyocyte hypertrophy.

### IKKi deficiency significantly activates AKT/GSK3β/mTOR/FOXO and NFκB signaling

**Figure 4:**
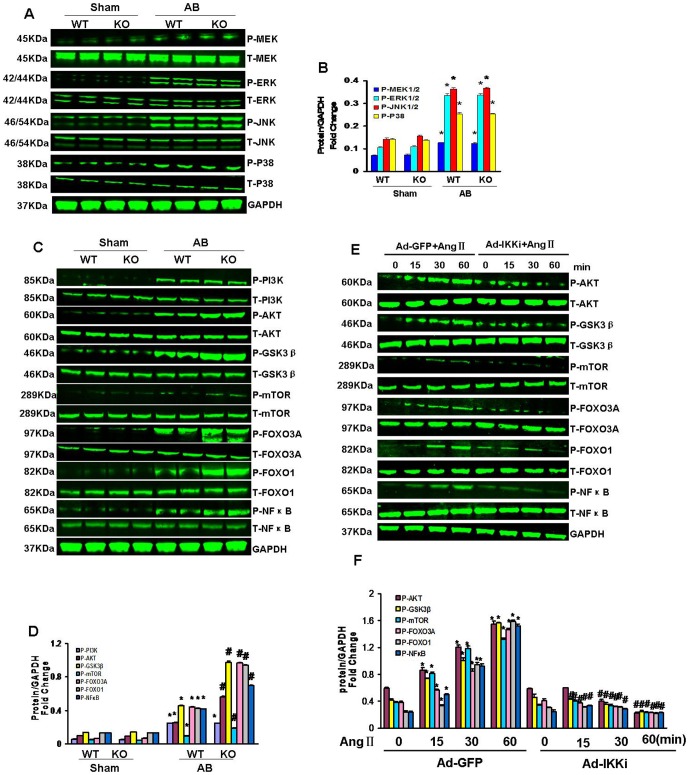
Effect of IKKi on the MAPK and AKT/GSK3β/mTOR/FOXO/NFκB signaling pathways. (A–D) Levels of total and phosphorylated MEK1/2, ERK1/2, JNK, P38PI3K, AKT, GSK3β, mTOR, FOXO and NFκB in the heart tissues of mice in the indicated groups (n = 6). A and C Representative blots. B and D Quantitative results. (E–F) Representative blots for total and phosphorylated AKT, GSK3β, mTOR, FOXO3A, FOXO1 and NFκB after treatment with Ang II for the indicated times in H9c2 rat cardiomyocytes infected with Ad-GFP or Ad-IKKi. E Representative blots. F, Quantitative results. *P<0.05 vs. WT/sham; # *P*<0.05 vs. WT/AB after AB.

To elucidate the molecular mechanism by which IKKi deficiency mediates the hypertrophic response, we examined the activation state of AKT and the expression of its downstream targets, including GSK3β, mTOR, forkhead box O3A (FOXO3A), forkhead box O1 (FOXO1) and NFκB using western blotting. The levels of phosphorylated AKT, GSK3β, mTOR, FOXO3A, FOXO1 and NF-κB were significantly increased in the hearts of the KO mice following pressure overload (Figures 4C, D). We then exposed cultured H9c2 cardiomyocytes infected with Ad-IKKi or Ad-GFP to 1 µMAngII. As shown in Figures 4E and F,AngII-stimulated AKT/GSK3β/mTOR/FOXO/NF-κB phosphorylation was attenuated by infection with Ad-IKKi.We further investigated the role of MAPK, another important signaling molecule that regulates the development of hypertrophy. However, the levels of phosphorylated MEK-ERK1/2, JNK, p38 and PI3K were similar between the AB groups (Figures 4A, B).

### Effects of IKKi deficiency on cardiac fibrosis induced by pressure overload

**Figure 5:**
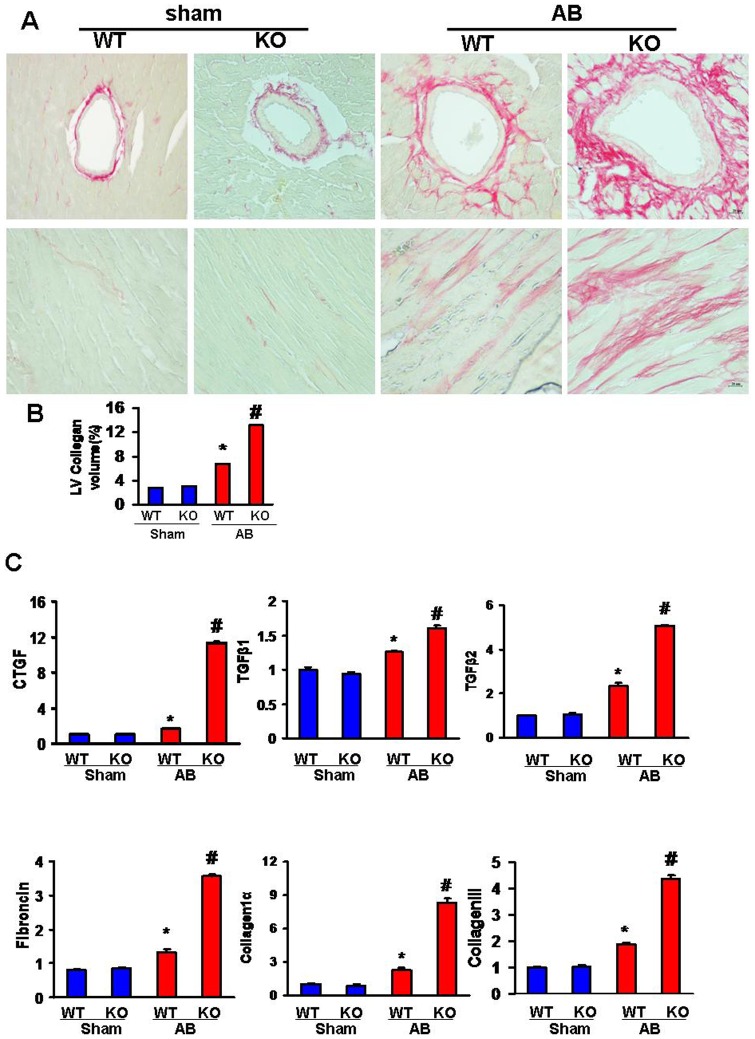
IKKi deficiency exacerbates the fibrotic response that is induced by pressure overload. A, Histological sections of the left ventricle were stained with picrosirius red in the indicated groups. B, The fibrotic areas from the histological sections were quantified using an image-analyzing system. C, The mRNA expression levels of collagen I, collagen III, fibronectin, TGF-β1, TGF-β2, and CTGF in the myocardium were obtained from the indicated groups using RT-PCR analysis. *P<0.05 vs. WT/sham; # *P*<0.05 vs. WT/AB after AB.

Fibrosis contributes to the phenotypic changes associated with the development of pathological cardiac hypertrophy. To detect the extent of fibrosis, we stained heart sections with PSR and found markedly increased perivascular and interstitial fibrosis and LV collagen volumes in the AB-induced KO and WT hearts compared with the sham-operated controls after 4 weeks. However, it should be noted that considerably increased cardiac fibrosis was also present in the KO mice (Figures 5A, B).

The observed interstitial fibrosis could be attributed to decreased collagen degradation or increased collagen synthesis. To evaluate the effect of IKKi on collagen synthesis, the mRNA expression levels of connective tissue growth factor (CTGF), transforming growth factor (TGF)-β1, TGF-β2, collagen I, collagen III, and fibronectin, which are known mediators of fibrosis, were analyzed. Increased expression levels of CTGF, TGF-β1, TGF-β2, collagen I, collagen III, and fibronectin were detected in the AB-induced KO mice (Figure 5C). These data suggest that IKKi deficiency promotes cardiac fibrosis.

### IKKi deficiency enhances cardiac apoptosis induced by press-ure overload

**Figure 6:**
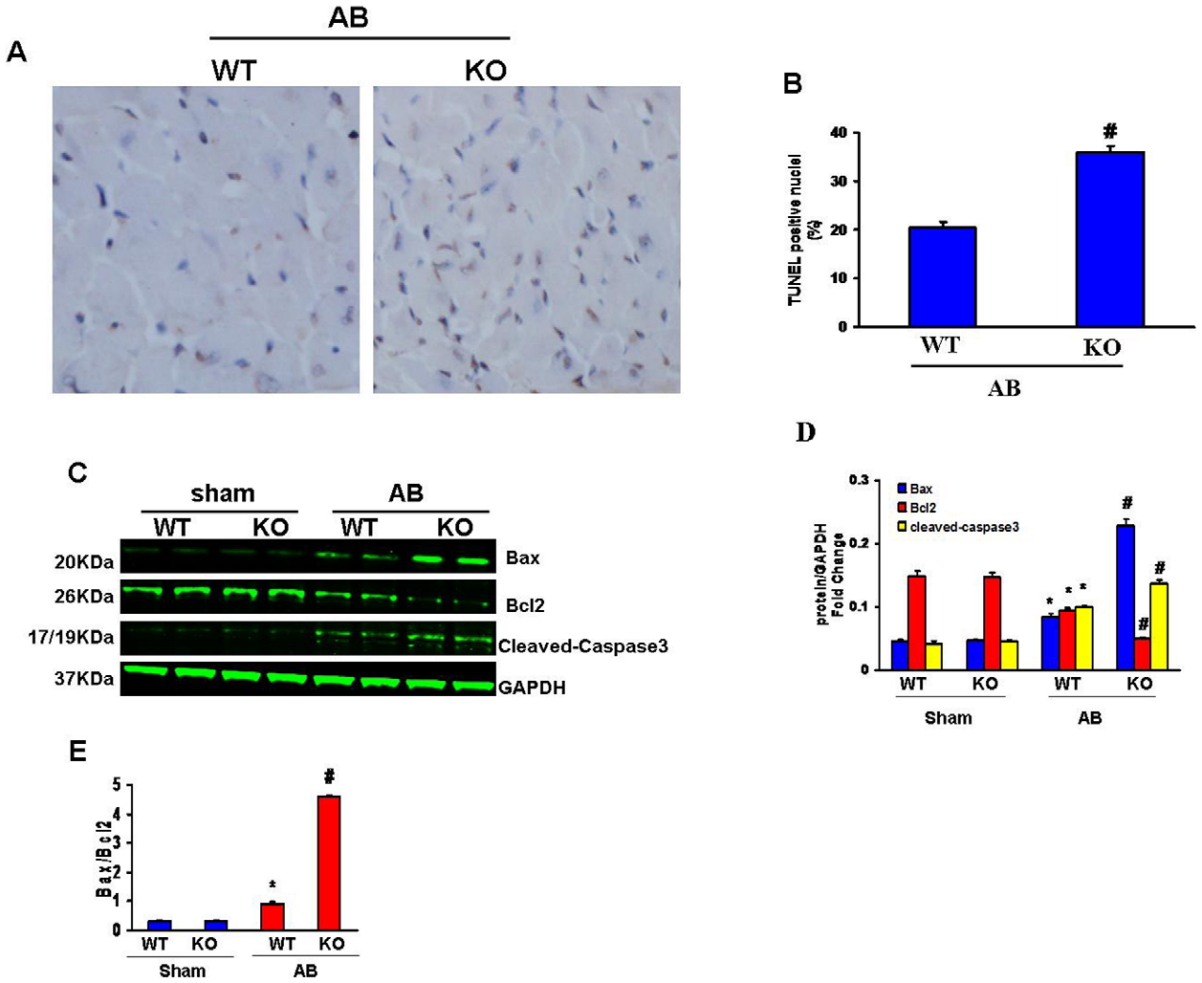
Effects of IKKi on cardiac apoptosis. A, TUNEL staining of AB mice at 4 weeks post-surgery. B, TUNEL-positive cells were quantified by the examination of 3000 nuclei from10 randomly selected fields per heart. # *P*<0.05 vs. WT/AB after AB.

To further explore the role of IKKi in hypertrophy, we assessed the apoptosis of myocytes after 4 weeks of AB using TUNEL assays. Apoptotic cells were detected in both KO and WT mice, and the fraction of apoptotic versus total cells was significantly higher in the pressure-overloaded hearts of the KO mice (Figures 6A,B). Furthermore, the levels of cleaved caspase 3, Bax (proapoptotic) and Bcl2 (antiapoptotic) proteins were assessed. The Bax protein level was increased and the Bcl2 protein level was decreased in the pressure-overloaded hearts of the KO mice, indicating a reduced Bcl2/Bax ratio. The level of cleaved caspase-3 was higher in the KO mice in response to AB (Figures 6C–E). These findings indicate that IKKi is involved in apoptosis in hypertrophic hearts subjected to pressure overload.

## Discussion

Cardiac hypertrophy is characterized by the reactivation of fetal cardiac genes, increased cross-sectional areas of adult cardiomyocytes, and contractile dysfunction. In this study, we investigated the role of IKKi and its related molecular mechanisms in cardiac hypertrophy. The present study demonstrated that IKKi deficiency deteriorated cardiac hypertrophy and fibrosis. The important novel findings of our study are as follows: (1) IKKi expression is increased in AB-induced hypertrophic heart tissue; (2) IKKi deficiency provokes spontaneous hypertrophy; (3) IKKi deficiency promotes pathological hypertrophy and fibrosis; (4) IKKi overexpression markedly attenuates the hypertrophy of H9c2 rat cardiomyocytes induced by Ang II in vitro; (5) IKKi deficiency exacerbates cardiac remodeling by activating AKT and NF-κB signaling; and (6)IKKi deficiency is involved in apoptosis in hypertrophic hearts subjected to pressure overload

As a member of the IKK family, IKKi (inducible IκB kinase), which is also known as IKKε, is involved in the activation of transcription factors [25]. Although IKKi is constitutively expressed in T cells, its expression is mainly regulated by NF-κB in other cell types [26]. Our data demonstrate that IKKi expression is significantly elevated in AB-induced hypertrophic heart tissues, which suggests that it is involved in promoting the development of cardiac hypertrophy and remodeling. This hypothesis is consistent with some studies involving other IKK family members, such as IKKβ [18].

The molecular mechanism by which IKKi affects cardiac remodeling remains unclear. To address this issue, we analyzed the activation of hypertrophic signaling pathways in AB mice. Pivotal signaling pathways that functioned in the pathogenesis of cardiac hypertrophy, including the mitogen-activated protein kinases (MAPKs) and AKT pathways, were assessed [10], [27], [29], [30], [31]. The downstream targets of AKT include GSK3β, mTOR,FOXO transcription factors and NFκB, all of which are involved in cardiac hypertrophy [10], [27], [29], [30], [31], [32], [33]. In this study,AKT phosphorylation was significantly enhanced in response to hypertrophic stimuli in the KO mice compared with WT mice. Consistent with the observed increase in AKT activity, hypertrophic stimuli caused increased levels of phosphorylated the GSK3β^Ser9^ and FOXO transcription factors at AKT phosphorylation sites (reducing their anti-hypertrophic effects) and increased activation of mTOR in the IKKi-deficient mice compared with WT mice. However, IKKi did not influence the phosphorylation of ERK1/2, JNK1/2, p38, MAPK or PI3K. Therefore, our study demonstrates that the AKT and NF-κB signalling is a critical pathway by which IKKi influences cardiomyocyte growth. Furthermore, we demonstrated that IKKi overexpression markedly inhibited AKT and NF-κB signaling in cultured cardiomyocytes stimulated by Ang II.However, the mechanism by which IKKi specifically activates AKT signaling remains unknown. In accordance with our findings, TRAF-associated NFκB activator-binding kinase1 (TBK1), another IκB kinase-related kinase, which exhibits 49% identity and 65% similarity to IKKi, another member of the IKK family, has been shown to control the activation of AKT [34], [35]. As a ligand for integrins, the absence of IKKi may be compensated for by TBK1,thus modulating integrin signaling or a specific integrin complex in a manner that specifically regulates AKT. Further experiments are needed to determine the molecular signaling mechanism by which IKKi regulates AKT. It is worth noting that the AKT pathway is a non-specific pathways, and a change in the AKT pathway is likely to be an indicator rather than a true determinant and a pharmacological target.

Fibrosis is an important contributor to the development of cardiac dysfunction in diverse pathological conditions. Fibrosis involves the progressive over-accumulation of extracellular matrix (ECM) (which surrounds and interconnects cells, is present in the myocardial wall and provides a scaffold for both myocytes and non-myocytes) components in cardiac muscle [36]. The major ECM proteins (type I and III collagens) are increasingly synthesized in the heart in response to pressure overload stimuli [36]. Moreover, the increased expression of TGF-β1 parallels the perivascular and myocardial interstitial fibrotic changes [37]. TGF-β and CTGF also modulate the proliferation of fibroblasts [30]. The present study has shown for the first time that IKKi deficiency leads to increased collagen deposition after AB and increased mRNA levels of CTGF, TGFβ and collagen I and III mRNA. These results suggest that IKKi deficiency promotes fibrosis and cardiac remodeling by enhancing collagen synthesis and up-regulating fibrotic mediators.

Cardiac myocyte apoptosis is also a critical factor during the transition from compensatory cardiac hypertrophy in response to pressure overload to heart failure [38].The present study showed increased apoptosis in the pressure-overloaded hearts of the KO mice compared with the WT mice. Furthermore, our results also demonstrated a significant increase in the of Bax-to-Bcl2 expression ratio and the activation of caspase-3 in the hearts of the KO mice after AB. Taken together,these data indicate that IKKi deficiency could influence apoptosis by affecting apoptosis-regulating proteins.

The roles of IKKi in the cardiac hypertrophic response to pressure overload have not been described previously. We are one step closer to elucidating the IKKi-related mechanisms that are associated with the development of cardiac hypertrophy,fibrosis and apoptosis. IKKi protects against hypertrophy via negative feedback of the AKT and NF-κB signaling pathway and concurrently regulates collagen deposition and fibrotic mediators. Taken together, our findings provide a rationale for further studies on the potential therapeutic benefits of IKKi in cardiovascular disease.
